# Oviposition Preference for Young Plants by the Large Cabbage Butterfly (*Pieris brassicae*) Does not Strongly Correlate with Caterpillar Performance

**DOI:** 10.1007/s10886-017-0853-9

**Published:** 2017-06-15

**Authors:** Minghui Fei, Jeffrey A. Harvey, Yi Yin, Rieta Gols

**Affiliations:** 1 Department of Terrestrial Ecology, Netherlands Institute of Ecology, Wageningen, The Netherlands; 2 Department of Ecological Sciences, Section Animal Ecology, VU University Amsterdam, Amsterdam, The Netherlands; 3 Laboratory of Entomology, Wageningen University and Research, Wageningen, The Netherlands

**Keywords:** Brassicaceae, Butterfly oviposition choice, Glucosinolates, Insect herbivores, Insect performance, Plant development, Plant ontogeny, Primary metabolites, Secondary metabolites

## Abstract

The effects of temporal variation in the quality of short-lived annual plants on oviposition preference and larval performance of insect herbivores has thus far received little attention. This study examines the effects of plant age on female oviposition preference and offspring performance in the large cabbage white butterfly *Pieris brassicae*. Adult female butterflies lay variable clusters of eggs on the underside of short-lived annual species in the family Brassicaceae, including the short-lived annuals *Brassica nigra* and *Sinapis arvensis,* which are important food plants for *P. brassicae* in The Netherlands. Here, we compared oviposition preference and larval performance of *P. brassicae* on three age classes (young, mature, and pre-senescing) of *B. nigra* and *S. arvensis* plants. Oviposition preference of *P. brassicae* declined with plant age in both plant species. Whereas larvae performed similarly on all three age classes in *B. nigra,* preference and performance were weakly correlated in *S. arvensis*. Analysis of primary (sugars and amino acids) and secondary (glucosinolates) chemistry in the plant shoots revealed that differences in their quality and quantity were more pronounced with respect to tissue type (leaves vs. flowers) than among different developmental stages of both plant species. Butterflies of *P. brassicae* may prefer younger and smaller plants for oviposition anticipating that future plant growth and size is optimally synchronized with the final larval instar, which contributes >80% of larval growth before pupation.

**Electronic supplementary material:**

The online version of this article (doi:10.1007/s10886-017-0853-9) contains supplementary material, which is available to authorized users.

## Introduction

The suitability of a food plant for growth and development of herbivorous insects is generally determined by a combination of sufficiently high concentrations of primary metabolites and low concentrations of secondary metabolites (Scriber and Slansky 1981). Primary metabolites, such as proteins, especially carbohydrates, and fatty acids are essential for the growth, development, and reproduction of all living organisms (Schoonhoven et al. 2005). Given its central role in most metabolic processes, nitrogen is considered to be of critical importance for the growth and development of insects and is often limiting in plant tissues reducing the quality of plants as food (Lawton and McNeill 1979; Mattson 1980; Scriber and Slansky 1981). Secondary metabolites often play a defensive role in resistance against insect herbivores (Fraenkel 1959; Iason et al. 2012; Mithöfer and Boland 2012; Schoonhoven et al. 2005). Resistance may involve traits that deter oviposition or feeding and interfere with the insect’s physiology once tissues have been ingested (Schoonhoven et al. 2005). However, most insect herbivores are dietary specialists and thus feed on only a few species or closely related plants that produce phylogenetically conserved secondary metabolites (Loxdale et al. 2011; Schoonhoven et al. 2005). Consequently, many insect herbivores have evolved various adaptations to deal with adverse phytochemicals (Despres et al. 2007; Nishida 2002; Winde and Wittstock 2011) and many species even use certain phytochemicals for host plant recognition (Renwick 1989).

The production of primary and secondary metabolites is largely genetically controlled and often varies with plant age and in response to biotic and abiotic factors such as rainfall, temperature and insect feeding and pathogen attack (Barton and Koricheva 2010; Karban and Baldwin 1997; Schoonhoven et al. 2005). Nitrogen demands (and N levels) are often higher in young growing tissues of plants and decline with age (Mattson 1980). Concentrations of secondary metabolites in plant tissues are also dynamic and can change with time. Using meta-analysis, Barton and Koricheva (2010) reviewed ontogenetic changes in plant secondary metabolites across a range of plant taxa and found that constitutive concentrations of all types of secondary metabolites significantly increased from the seedling to the adult stage in both herbaceous and woody plants.

Adult females of insect herbivores must find suitable food plants for their offspring, and these plants are often found in habitats that vary in structural and chemical complexity. When the temporal availability and quality of suitable food plants is highly predictable, searching for oviposition sites is often closely synchronized with these parameters (Wolda 1988). For example, larval feeding stages of monophagous insects that have only one generation per year (i.e., are univoltine) usually occur only when suitable plant tissues, such as young leaves, are available (van Asch et al. 2010). In contrast, when adult females of multivoltine herbivorous insects that feed on short-lived herbaceous plant species are searching for oviposition sites, they are faced with a range of challenges. For example, different generations have to find suitable food plant species that may differ in quality due to differences in plant age, which can potentially have significant effects on the development, survival and fitness of their progeny.

According to the preference-performance hypothesis, females should maximize their fitness by laying eggs on plants on which the offspring perform the best (Jaenike 1990; Mayhew 1998). However, this hypothesis ignores the fact that the relationship between female preference and offspring performance may also be influenced by other ecological factors such as predation risk, the dietary breadth of the herbivores and food plant availability at the time of oviposition (Gripenberg et al. 2010). These other considerations may explain why the evidence supporting the preference-performance hypothesis is variable (Mayhew 2001; Scheirs et al. 2005). When the quality of host plants varies unpredictably over time, females may fail to evolve the ability to choose the plant type that would be most suitable for development of their offspring (Cronin et al. 2001; Gripenberg et al. 2010). Multivoltine insect herbivores may be forced to use different host plant species over the course of a growing season, depending on what is available at a specific time and their performance may be constrained by temporal and species-specific variation in host plant quality as well as quantity (in the case of gregarious species) (Fei et al. 2014; Fei et al. 2016a; Gols et al. 2007).

In this study, we examine female oviposition preference and offspring performance of the large cabbage white butterfly *Pieris brassicae* L. (Lepidoptera: Pieridae) in relation to plant ontogeny. Larval stages of this herbivore feed only on plants that contain glucosinolates, defensive secondary metabolites characteristic of species in the plant family Brassicaceae (Fahey et al. 2001). Adult females and larvae of *P. brassicae* use glucosinolates as oviposition and feeding stimulants, respectively (Renwick 2002; van Loon et al. 1992). Most of the host plant species of *P. brassicae* are short-lived annuals that are present in the field for only two to three months (Feltwell 1982). These include cabbage crops and mustard oil species on which *P. brassicae* is considered an important pest species across much of Eurasia (Feltwell 1982). In the Netherlands, *P. brassicae* has two to three generations per year. In relation to the phenology of the herbivore and its potential host plants, amongst others, the charlock mustard *Sinapis arvensis* L., which grows in late spring and early summer and the black mustard, *Brassica nigra* L., which grows in middle to late summer, are considered important food plants for this herbivore. These ephemeral plant species often grow in dense populations, which is important because *P. brassicae* lays eggs in clutches of 30–100 eggs on a single plant and at least several plants are required to support the complete development of an entire brood (Fei et al. 2016a). Furthermore, previous work has shown that *P. brassicae* caterpillars not only feed on the leaves, but that older larvae move up the plants and feed on the flowers (Smallegange et al. 2007). When caterpillars were only allowed to consume flowers, they developed faster and obtained a higher final caterpillar mass compared to conspecifics that were restricted to feed on leaves (Smallegange et al. 2007). These results suggest that the flowers are of higher nutritional quality than the leaves.

In the field, when female *P. brassicae* butterflies are searching for oviposition sites, they often encounter plant individuals of variable age and potentially quality. These plants may also vary considerably in size, as *B. nigra* plants grow as high as 2 m or more, depending on soil quality, access to light, competition with other plants and nutrient availability. The aims of this study are to 1) investigate whether female butterflies distinguish between different developmental stages of the two host plant species for oviposition and 2) to determine whether female butterflies lay their eggs on plants in the developmental stage that is best for growth and development of their offspring. Nutritional quality and levels of defensive chemistry are usually higher in young developing leaves than in old mature leaves (Bowers and Stamp 1993; Scriber and Slansky 1981). Also, glucosinolate concentrations are generally higher in newly developed leaves than in older leaves (Gols et al. 2007). As *P. brassicae* caterpillars are specialist herbivores on several species of brassicaceous plants including the two species studied here, we predict that larval development will not be affected by variable concentrations of glucosinolates. Instead, larval performance will be better on younger plants that are of higher nutritional quality in terms of primary metabolites than on older plants. To link larval performance with qualitative characteristics of the plant tissues, we analyzed and quantified primary (amino acids and sugars) and secondary metabolites (glucosinolates) in tissues collected from plants in different developmental stages and correlated these with larval performance variables using comprehensive multivariate statistics.

## Methods and Materials

### Plants and Insects

*Sinapis arvensis* and *Brassica nigra* grow in disturbed sites, such as along river flood plains and in areas where soil is disturbed and nutrient availability is high. In the Netherlands, these two plant species are very important food plants for *P. brassicae*. Seeds of both species were collected from several plants (> 15) in wild populations growing naturally near Wageningen, the Netherlands. Seeds were germinated and seedlings were subsequently transferred to 1.1-L pots filled with peat soil (‘Lentse potgrond’ no.4; lent, The Netherlands). Plants were grown in a greenhouse at 21 ± 2 °C (day) and 16 ± 2 °C (night), 50% r.h., and a photoperiod of at least 16 hr. If the light dropped below 225 μmol photons m^2^ s^−1^ during the 16-h photoperiod, supplementary illumination was applied by sodium lamps. The plants were watered twice a week during the first 3 weeks of development and watering was then gradually increased to once daily. When the plants were 3 weeks old, they were fertilized once a week with Hoagland solution which was applied to the soil. Watering and fertilization continued during the experiments. This fertilization regime is necessary to sustain normal growth of these fast growing plants in small pots.

To investigate the effect of plant developmental stage (and size) on insect preference and performance, plants of different age classes were reared by sowing the seeds and growing the plants at different time points. Because *S. arvensis* has a shorter life cycle than *B. nigra*, different age classes were used for the two species. Seeds of *S. arvensis* were germinated at two-week intervals and plants used in the experiment were 3, 5 and 7 weeks old, respectively. In the greenhouse, three-week old *S. arvensis* plants are in the transition between the vegetative and reproductive developmental phase; plants are still developing leaves, but flower buds are starting to develop as well. Five-week old *S. arvensis* plants are flowering and the leaves are still succulent (mid-reproductive phase), whereas seven-week old plants are still flowering, but the leaves are starting to senescence (late-reproductive phase). Seeds of *B. nigra* were germinated at three-week intervals and plants used in the experiments were 3, 6 and 9 weeks old, respectively. Three-week old *B. nigra* plants are still in the vegetative developmental phase, six-week old plants are in the late vegetative/early reproductive phase, whereas nine-week old plants are fully flowering and still have green leaves (=mid-reproductive phase).

Cultures of *P. brassicae* were maintained in a climate room at 22 ± 2 °C, 50–70% r.h. and a photoperiod of at least 16 h. *P. brassicae* which had been collected from the field in the previous summer were reared on Brussels sprout plants (*B. oleracea* var. gemmifera, cv. Cyrus) for approximately 10 generations at the Laboratory of Entomology, Wageningen University.

### Experiment 1. Butterfly Oviposition Preference in Relation to Plant Ontogenetic Stage

Larvae of *P. brassicae* were reared on Brussels sprouts and pupae were collected from the rearing cages and transferred to a clean cage (60 × 60 × 180 cm) placed in the experimental greenhouse. Newly emerged adult butterflies were provided with a (20%) honey solution applied to cotton wool in blue plastic caps to promote feeding activity. The butterflies were 3–5 days old when used in the choice bioassays.

Oviposition preference was determined in three-choice-experiments in tents (2 × 2 × 2 m) placed outside in the garden of the Netherlands Institute of Ecology on bare soil. Plants of the three different age classes, one plant from each class, were prepared as described in the *Plants and insects* section. Bioassays were performed for each plant species separately.

Single plants from the three different age classes were randomly placed in one of three corners of the tent. Unmated females only lay a few infertile eggs. As not all females in the rearing cage may have mated, one female and one male butterfly were released in the tent to allow additional mating. A bioassay was terminated when a female butterfly had laid her first egg clutch which was checked every hour. The age class of the plant on which the eggs were laid was recorded. The bioassay was repeated at least 50 times for each plant species. Bioassays were conducted from June to August. New plants and butterflies were used for each replicate.

Plants of the three age classes differed in size. To determine whether differences in size of the plants influenced oviposition decisions, we also measured total leaf surface area and plant height of at least ten plants per age class. For leaf surface area measurements, all the leaves from single plants were collected and scanned using a photo scanner (Perfection4990; Epson, Japan). The leaf surface area was determined using the software WinFOLIA (Regent Instruments, Sainte-Foy, Canada).

### Experiment 2a. Ontogenetic Effects of Plant Quality on Herbivore Performance

To obtain larvae for the performance bioassay, plants of the various developmental stages were placed in the rearing cage with *P. brassicae* butterflies. The females used for oviposition here were not the same as used in the oviposition bioassays. Since *P. brassicae* eggs need about one week to hatch under the greenhouse conditions used in this study, *B. nigra* and *S. arvensis* plants were prepared one week before they were used in the experiments. Female butterflies were allowed to oviposit on these plants for 24 h. The following day, the plants were removed from the butterfly cage and transferred to the experimental greenhouse. Larvae hatching from the eggs were allowed to feed upon their natal plants until they reached the second instar. Egg hatching was close to 100% and mortality of first instar caterpillars was very low (< 5%). Using a fine paint brush, five cohorts (= replicates) of 20 randomly selected 1-day old L2 larvae were transferred to cages containing four plants that were in the same developmental stage as the one on which the caterpillars had hatched and had been feeding previously. As young *P. brassicae* caterpillars prefer to feed gregariously, they were introduced onto a single fully developed leaf of one plant in a cage.

Depending on the developmental stage of the plants, we used differently sized cages (40 × 40 × 60 cm and 60 × 60 × 180, respectively, Vermandel, Hulst, The Netherlands). In order to exclude a possible effect of temperature differences in the two differently-sized cages, we measured the temperature in the middle of the cages twice a day. There was no significant difference in temperatures between the two cage sizes. Caterpillars were allowed to move and feed freely on the plants within a cage until they pupated. Pupation was recorded daily and pupae were collected and weighed on an analytical balance (0.1 mg accuracy). During the experiment, extra plants of the same age as the plants in the cage were added when needed (that is when more than 50% of the plant tissues had been consumed). As proxies for insect performance, we measured insect development time from egg hatching to pupation, pupal fresh mass and survival to pupation. The conditions were the same as for plant growth.

### Experiment 2b. Plant Ontogenetic Changes in Chemical Profiles

During the larval performance bioassays, plant tissues were collected for analysis of primary (sugars and amino acids) and secondary metabolites (glucosinolates) from both *B. nigra* and *S. arvensis* plants in the three age classes. Tissues were sampled from the plants when the caterpillars had been feeding on them for 9 days. Caterpillars were removed before sampling. Leaf tissues were collected by excising five 7 mm (diameter) discs from each of four fully developed green leaves of two plants within each cage. As *P. brassicae* caterpillars feed on both leaves and flowers (Smallegange et al. 2007), we also collected flowers for chemical analysis. Eight stems with flowers were cut off the same plants from which leaf tissues had been collected. In addition, leaf and flower tissues were sampled from control plants in the same three age classes that had not been exposed to herbivory, but were otherwise treated the same as those exposed to caterpillar feeding. Tissues were sampled in the morning between 10:00 and 12:00. Leaf discs and flowers, respectively, were pooled per cage in tinfoil and frozen in liquid nitrogen immediately after sampling and stored at −80 °C until further processing. Samples were freeze-dried and pulverized with a grinding machine (Retsch GmbH, type MM 301).

For quantification and identification of soluble sugars, amino acids, and glucosinolates, one global extraction was conducted for each sample. Approximately 50.0 mg of finely ground plant material was suspended in 1.0 ml 70% MeOH in water (vol/vol), vortexed, and immediately boiled for 5 min to inactive enzymes. Eppendorf tubes containing the sample were incubated in an ultrasonic bath for 15 min and then centrifuged for 10 min at 10000 *g*. The extraction was repeated with the pellet, omitting the boiling step. Both supernatants were combined per sample and supplemented with 70% MeOH to a final volume of 2.0 ml. This ‘stock’ extract was stored at −20 °C until further analysis.

Soluble sugars were separated by ion-exchange high-performance liquid chromatography (HPLC) with a CarboPac PA1 main column (2 × 250 mm) and a CarboPac PA1 guard column (2 × 50 mm). Sugars were separated using an isocratic gradient mixture of 10% 1 M NaOH in water at a flow rate of 0.25 ml min^−1^ and a column temperature of 20 °C (for more details see van Dam and Oomen (2008)). A reference solution containing 54.9 μM sorbitol and mannitol, 29.2 μM trehalose, sucrose and melibiose, and 55.5 μM glucose and fructose was diluted to obtain calibration standards of 2.5, 5 and 7.5 ppm, respectively, for construction of a reference curve. Quantification was based on these reference curves.

Amino acids were analyzed on an Ion-exchange HPLC with an AminoPac PA10 main column (2 × 250 mm) and an AminoPac PA10 guard column (2 × 50 mm) and were separated with a tenary gradient. The method is described in detail in van Dam and Oomen (2008). For quantification and identification a reference sample was prepared containing the Sigma AA-S-18 amino acid standard (Sigma, St Louis, MO, USA) supplemented with asparagine, glutamine and tryptophan each at a concentration of 2.5 mM ml^−1^.

For glucosinolate analysis, 1 ml of the stock was applied to a DEAE-Sephadex A25 column and desulfated with sulfatase (type H-1 from *Helix pomatia,* Sigma-Aldrich). Glucosinolates were separated by HPLC with an acetonitrile-water gradient (2–65% acetonitrile from 0 to 30 min; flow 0.75 ml min^−1^; column temperature; 40 °C) on a reversed phase Alltima C-18 column (150 × 4.6 mm). For detection of the glucosinolates a photodiode array detector was used set at 229 nm as the integration wavelength. The method is described in detail in van van Dam et al. (2004). For quantification, sinigrin was used as an external standard. Glucosinolate identification was based on their retention times and their UV spectra were compared to those of pure compounds provided by M. Reichelt (Max Planck Institute for Chemical Ecology, Jena, Germany) and a certified rapeseed standard (community Bureaus of Reference, Brussels, Belgium, code BCR-367 R).

### Statistical Analyses

Data were analyzed for the two plant species separately. To analyze butterfly oviposition preference, we used χ^2−^tests comparing the observed oviposition counts on the three age classes with an expected distribution of 1:1:1. When the test result was significant, we conducted pairwise χ^2−^tests with α = 0.05/3 to correct for type I errors (Bonferroni correction). Data on plant height and leaf area were analyzed using one-way ANOVA with plant developmental class as a fixed factor, followed by Tukey-Kramer multiple pair-wise comparison tests to reveal differences between means.

In the experiments measuring larval performance on plants belonging to different age classes, the five cages, each containing 20 caterpillars, served as experimental units. Therefore, we first calculated the mean values per cage for the two performance variables, pupal mass and egg-to-pupa development time. These mean values were then subjected to one-way ANOVA with plant developmental class as a fixed factor. Tukey-Kramer multiple comparison tests between means were conducted when the ANOVA models were significant. The ANOVA tests were carried out using SPSS (IBM SPSS, statistics version 19).

We used a multivariate statistical approach, i.e. constrained principal component redundancy analysis (RDA), to determine differences in plant chemistry among the three age classes, the two tissue types (leaves and flowers) and damage status (control or damaged by *P. brassicae* feeding). The data were ‘constrained’ by classifying them according to tissue type, developmental stage and damage status. Multivariate partitioning of variance analysis was used to determine how much of the variation in the chemistry variables was uniquely explained by each of the three factors. The multivariate counterpart of the ordinary F-ratio in univariate statistics was calculated using the sums of squares totaled across all response variables to yield the H_0_ F-statistic, which is referred to as a pseudo F-statistic. Monte Carlo permutation (default setting of 999 permutations) tests were used to determine whether model terms (tissue type, developmental stage, damage status) were significant or not (ter Braak and Šmilauer 2012). RDA was also used to analyze whether there is a significant correlation between compounds and herbivore fitness parameters (biomass and development time). For analysis of the chemical data we used the concentrations of the various sugars, amino acids, and glucosinolates, expressed as amount of chemical per unit dry weight of tissue and for the herbivore performance data we used the corresponding mean pupal weight and developmental time. Data (log-transformed and mean-centered) were analyzed for the two plant species separately. RDA analysis on all chemical data was performed in Canoco version 5.03 (ter Braak and Šmilauer, The Netherlands).

## Results

### Experiment 1: Butterfly Oviposition Preference in Relation to Plant Ontogenetic Stage

**Fig. 1:**
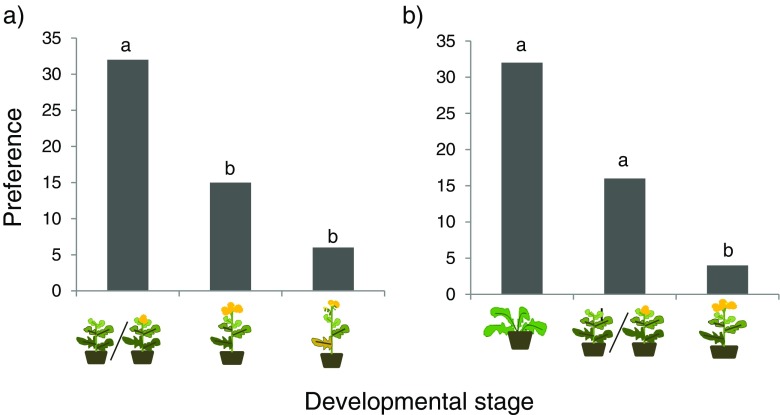
Counts of oviposition events by *Pieris brassicae* butterflies on *Sinapis arvensis* (**a**) and *Brassica nigra* (**b**) that were in different stages of development. Represents vegetative phase,  represents late vegetative to early reproductive phase,  represents mid-reproductive phase,  represents late-reproductive phase. Bars present the cumulative counts of oviposition choices (*n* = 53 for *S. arvensis* and *n* = 52 for *B. nigra*), bars with the same letter are not significantly different (pairwise χ^2^-tests with a Bonferroni correction for multiple comparisons)

All eggs were laid on the leaves of the plant and not on the flowers when they were present. Female *P. brassicae* butterflies clearly discriminated between the three age classes of *S. arvensis* (*χ* ^*2*^_2_ = 19.7, *P* < 0.001) and *B. nigra* (*χ* ^*2*^_2_ = 22.8, *P* < 0.001), respectively, when deciding where to oviposit (Fig. 1). Oviposition preference order declined with plant age for both plant species (Fig. 1). Females preferred to lay eggs on plants in the vegetative or early reproductive developmental phase over plants that were in the middle to late reproductive phase.

**Fig. 2:**
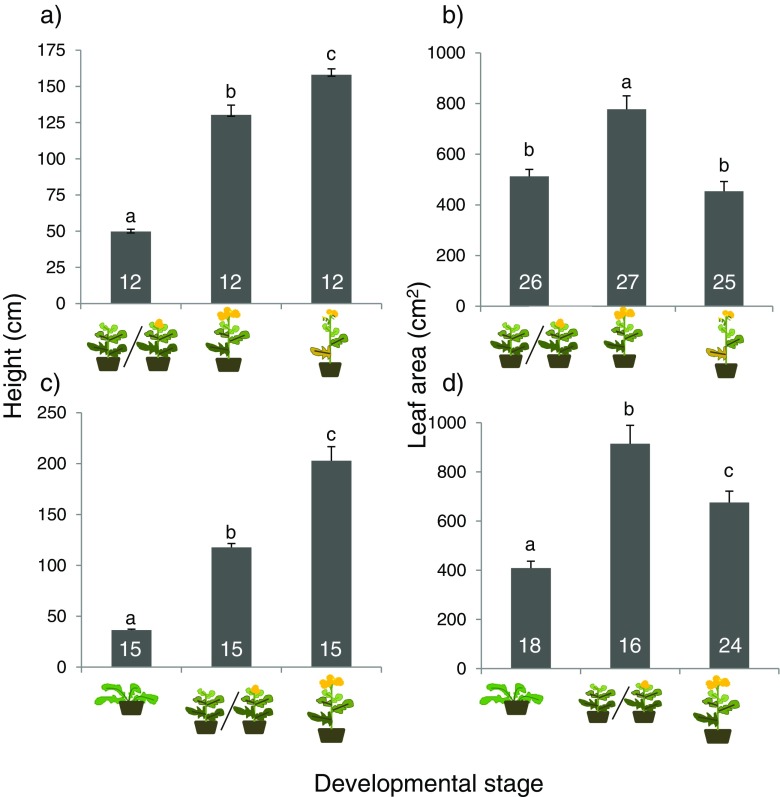
Effect of developmental stage on plant height (**a**, **c**) and total leaf surface area (**b**, **d**) in *Sinapis arvensis* (**a**, **b**) and *Brassica nigra* (**c**, **d**).  Represents vegetative phase,  represents late-vegetative to early-reproductive phase,  represents mid-reproductive phase,  represents late-reproductive phase. Bars present the means + SE (sample sizes are given in the bars) and bars with the same letter are not significantly different (Tukey-Kramer tests for multiple comparisons among means, α = 0.05)

Plant height differed among the three age classes in both plant species (*S. arvensis*: F_2,35_ = 147, *P* < 0.001, Fig. 2a; *B. nigra*: *F*_2,44_ = 101, *P* < 0.001; Fig. 2c) and correlated positively with plant age. In addition, there was an effect of age class on total leaf surface area (*S. arvensis*, *F*_2,77_ = 17.7, *P* < 0.001, Fig. 2b; *B. nigra*, *F*_2,57_ = 21.7, *P* < 0.001, Fig. 2d). In *S. arvensis*, total leaf surface area was higher in the middle reproductive phase than in plants that were in the vegetative/early reproductive phase (leaves on these plants were still expanding) and late reproductive phase (leaves on these plants started to wilt and die) (Fig. 2b). In *B. nigra*, vegetative plants were still developing new leaves and total leaf area was lowest in these plants. Total leaf area was intermediate in plants in the mid-reproductive phase on which the older leaves started to wilt and die, and leaf area was highest in plants in the vegetative/early reproductive phase (Fig. 2d).

### Experiment 2a: Ontogenetic Effects of Plant Quality on Herbivore Performance

**Fig. 3:**
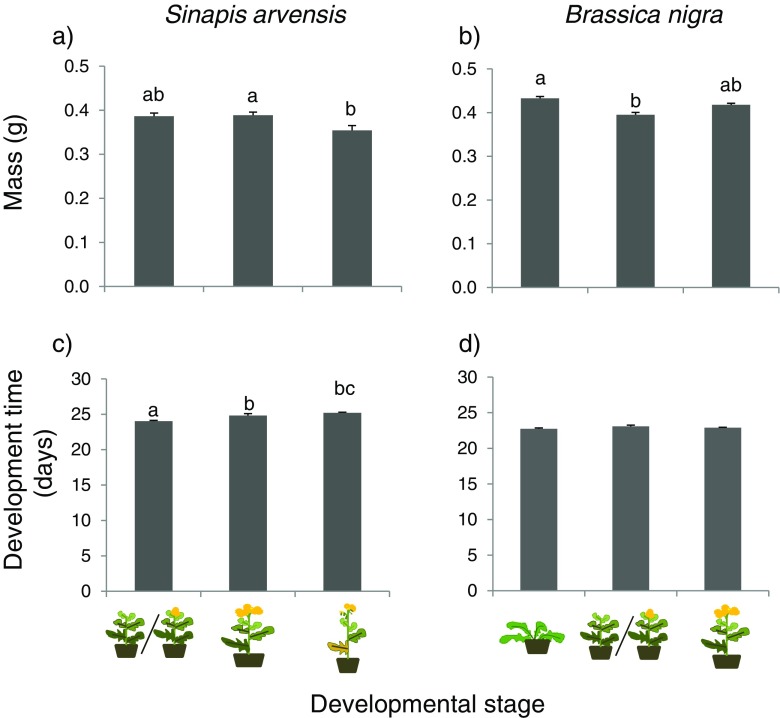
Pupal fresh mass (**a**, **b**) and egg-to-pupa development time (**c**, **d**) of *Pieris brassicae* reared on three developmental stages of *Sinapis arvensis* (**a**, **c**) and *Brassica nigra* (**b**, **d**).  Represents vegetative phase,  /  represents late-vegetative and early-reproductive phase respectively,  represents middle-reproductive phase, ` represents late-reproductive phase. Bars present the means + SE (*n* = 5 cages and 20 L2 caterpillars had been released in each cage) and bars with the same letter are not significantly different (Tukey-Kramer tests for multiple comparisons among means, α =0.05)

Both egg-to-pupa development time (*F*_2,14_ = 11.7, *P* = 0.002) and pupal mass (*F*_2,14_ = 5.07, *P* = 0.026) were affected by the age class of the *S. arvensis* plants on which the insects had been reared. Insects developed slower and developed into smaller pupae with increasing age of the plants (Fig. 3a and c).

The effect of plant developmental stage on food plant quality for *P. brassicae* was less pronounced in *B. nigra* than in *S. arvensis,* and affected only pupal mass (*F*_2,14_ = 18.3, *P* < 0.001, Fig. 3b) and not egg-to-pupa development time (*F*_2,14_ = 2.09, *P* = 0.17 Fig. 3d)*.* Pupae were heavier on plants in both the vegetative and mid-reproductive developmental phase than on plants in the late-vegetative/early reproductive phase (Fig. 3b).

### Experiment 2b: Plant Ontogenetic Changes in Chemical Profiles

**Fig. 4:**
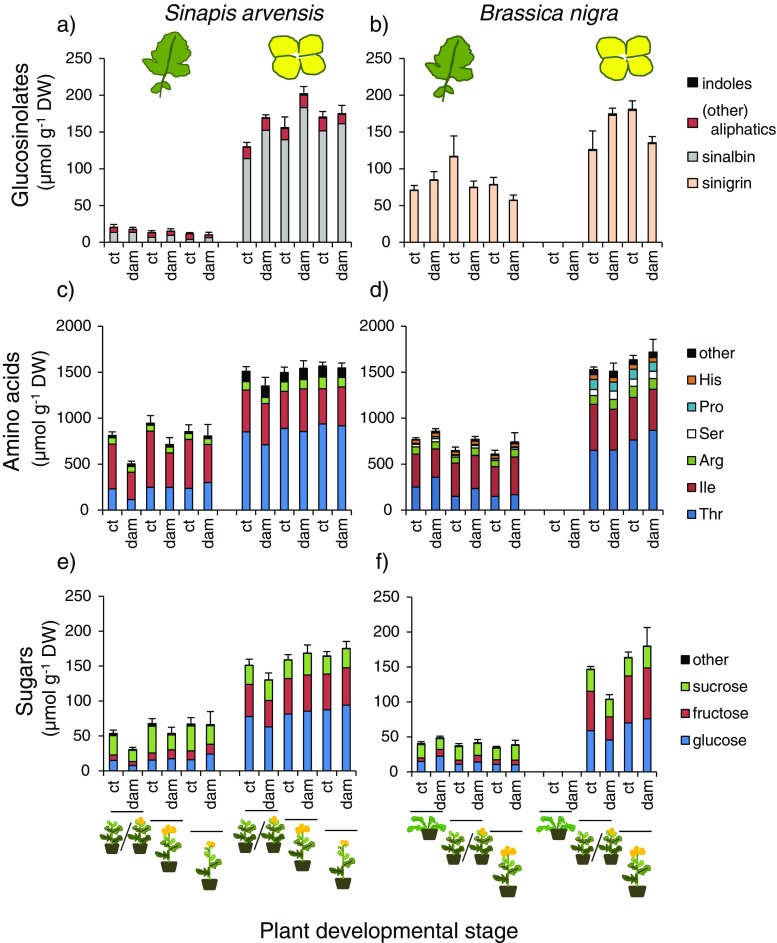
Total concentrations (mean + SE) of glucosinolates (**a**, **b**) amino acids (**c**, **d**) and sugars (**e**, **f**) in leaf and flower tissues of *Sinapis arvensis* (**a**, **c**, **e**) and *Brassica nigra* (**b**, **d**, **f**) that were sampled from plants in three different developmental stages.  Represents vegetative phase,  represents late vegetative to early reproductive phase,  represents mid-reproductive phase,  represents late-reproductive phase. In each panel metabolite concentrations in μmol g^−1^ dry weight (DW) are given for the leaves (*left*) and flowers (*right*) and for undamaged control plants (=ct) and plants exposed to *Pieris brassicae* feeding (=dam). Colours within bars refer to different compounds or classes of compounds (glucosinolates). Each bar represents the mean + SE (5 biological replicates) of pooled samples of 5 discs from each of 4 leaves or 8 flowers collected from two plants per replicate

**Fig. 5:**
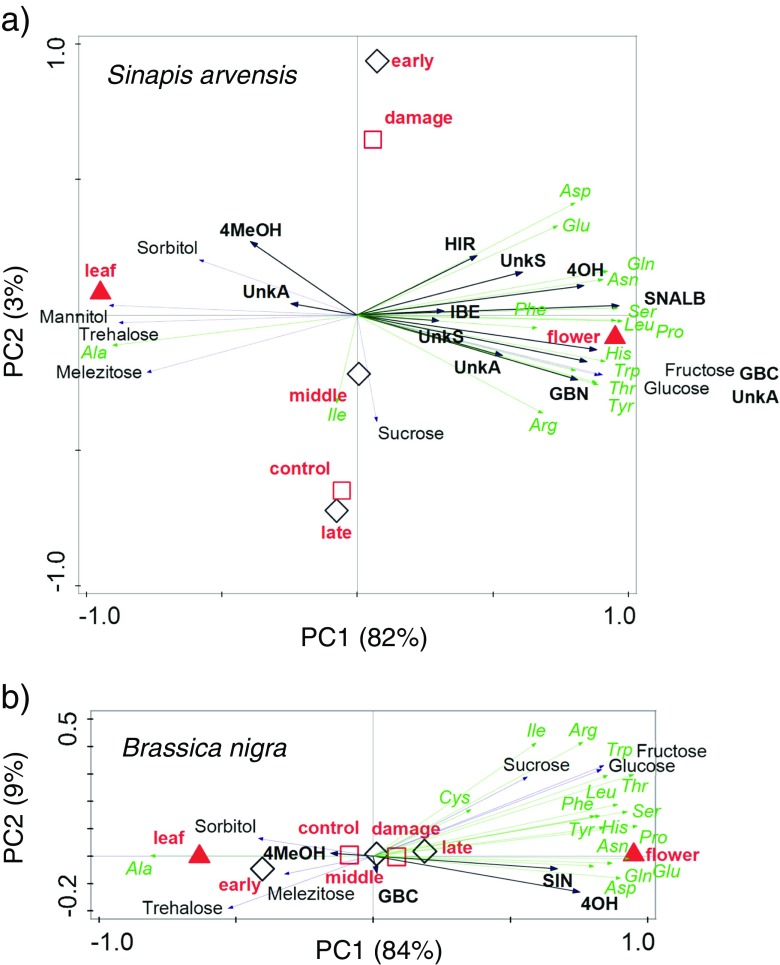
Redundancy analyses (RDA) on the chemical profiles (sugar, amino acid and glucosinolate (GS) concentrations) of *Sinapis arvensis* (**a**) and *Brassica nigra* (**b**) leaf and flower tissues. The statistics are given in Table 1. The centroids depict mean scores for the various classes; leaf and flower tissues (*red triangles*), plant developmental stages (*black open diamonds*) and herbivore-damaged or control tissues (*red open squares*). The loadings (contribution of the chemical variables) are given in *solid black lines* and *arrows* for the GS, dotted green lines and arrows for the amino acids, and *black dotted lines* for the sugars. The distance from the axes origin to each of the arrows indicates the magnitude of the contribution of the variable to the principal components. For the amino acids (*green*) the international IUPAC three letter codes were used. Sugar names are in *black* and glucosinolate names are in *bold black font* where SIN = sinigrin (2-propenyl GS), GBN = glucobrassicanapin (3-butenyl GS), IBE = glucoiberin (3-methylsulfinylpropyl GS), HIR = glucohirsutin (8-methylsulfinyloctyl GS =, UnkA = unknown aliphatic GS (not further specified), UnkS = unknown sulfur containing GS, GBC = glucobrassicin (indol-3-ylmethyl GS), 4OH = 4-hydroxyglucobrassicin (4-hydroxyindol-3-ylmethyl GS), 4MeOH = 4-methoxyglucobrassicin (4-methoxyindol-3-ylmethyl GS), SNALB = sinalbin (4-hydroxbenzyl GS). In 5a, early represents late-vegetative/early reproductive phase, middle represents mid-reproductive phase, and late represents late-reproductive phase. In 5b, early represents early-vegetative phase, middle represents late-vegetative/early reproductive phase, and late represents middle-reproductive phase

In leaf and flower tissues of *S. arvensis* 12 different glucosinolates, 7 sugars and 16 amino acids were detected, whereas in *B. nigra* 5 glucosinolates, 6 sugars and 16 amino acids were detected. In both plant species the differences in chemical profiles were most prominent between the two tissue types (Figs. 4 and 5). In *B. nigra,* differences between leaves and flowers primarily affected the quantity of individual compounds and not their quality (presence/absence). In addition to quantitative differences, some metabolites were only detected in the flowers (leucine, tryptophan, 4-hydroxyglucobrassicin), and others (mannitol, melibiose, alanine) only in the leaves of *S. arvensis.* In both plant species, concentrations of many chemicals were higher in the flowers than in the leaves (Fig. 4), with the exception of the minor sugars, sorbitol, trehalose and melezitose, the amino acid, alanine and the indole glucosinolate 4-methoxyglucobrassicin, of which the concentrations were relatively higher in leaf tissues of both plant species (Fig. 5). Glucosinolate concentrations were also higher in the flowers than in the leaves, especially concentrations of the most dominant *g*lucosinolates. In *B. nigra*, sinigrin (*SIN*)*,* contributed >98% to the total glucosinolate content in both the flowers and leaves, and in *S. arvensis* sinalbin (*SNALB*) contributed on average 60% and 90*%* to the glucosinolate content of the leaves and the flowers, respectively. Compared to differences related to tissue type, developmental stage and herbivory explained relatively little of the variation in chemistry attributes. Multivariate statistics confirmed this pattern (Table 1). For both plant species more than 80% of the variation in chemistry was attributed to differences between leaves and flowers and only a few percent was explained by plant developmental stage (< 4%) and whether plant tissues had been exposed to herbivory or not (< 2%).

**Table 1:** Multivariate RDA statistics using chemical data of flowers and leaves together or separately

Factors	Tissue type			Developmental stage			Herbivory (yes/no)			
	F^a^	*P* ^b^	% expl^c^.	F	*P*	% expl.	F	*P*	% expl	PCs^d^
Plant species										
*S. arvensis*	312	≤ 0.001	85.5	4.4	≤ 0.001	1.9	7.3	≤ 0.001	1.7	3
Flowers				3.3	≤ 0.001	13.2	4.1	0.003	9.0	2
Leaves				5.4	≤ 0.001	20.1	8.6	≤ 0.001	17.9	2
*B. nigra*	233	≤ 0.001	81.6	5.6	≤ 0.001	3.1	3.7	0.02	0.9	1
Flowers				1.0	0.40	0.2	0.8	0.64	0	0
Leaves				9.5	≤ 0.001	28.4	17.0	≤ 0.001	27.7	2

^a^Pseudo H_0_ F-statistic used in the Monte Carlo permutation test

^b^Significance level of the Monte Carlo permutation test

^c^Adjusted percentage of the explained variation

^d^PCs are the number of significant principal components (*P* < 0.05) in each model

Additional multivariate statistical models analyzing flower and leaf data separately with only developmental stage and herbivory status as explanatory variables revealed that these factors affected chemical profiles of the leaves stronger than those of the flowers, as the variation explained by these factors was higher for the leaves than for the flowers (Table 1, the graphs are given in the Supplementary Material). Chemical profiles of the flowers of *B. nigra* could not be separated according to developmental stage or herbivory status. In *B. nigra*, younger leaf tissues tended to have higher levels of most amino acids and the sugars, sorbitol and glucose, whereas levels of trehalose and alanine tended to be higher in the leaves of young and middle-aged *B. nigra* plants. Levels of the amino acids glutamine, tyrosine and tryptophan were relatively higher in damaged than in control *B. nigra* leaf tissues. The effects of developmental stage and damage status on glucosinolate concentrations were relatively small in this plant species. Contrastingly, in the leaves of *S. arvensis*, sinalbin, the dominant glucosinolate in this plant species, decreased with plant age, whereas in the flowers the opposite pattern was found for this compound. Concentrations of sinalbin and the indole glucosinolate 4-methoxyglucobrassicin were also higher in damaged than in control tissues. Concentrations of most sugars increased with age of the plants, both in the flowers and the leaves. The changes in amino acid concentrations in relation to plant age were more idiosyncratic. Sorbitol glutamine, and aspartic acid were relatively high in damaged leaves, whereas melibiose, sucrose and isoleucine were relatively high in undamaged control leaf tissues. Tryptophan was relatively high in control tissues and sinalbin was relatively high in damaged tissues.

### Linking Insect Performance to Plant Chemistry

For *S. arvensis*, significant correlations were found between insect performance variables and plant chemistry for both the leaves (RDA, *P* = 0.004) and the flowers (RDA, *P* = 0.048). For *B. nigra*, significant correlations were found between insect performance variables and plant chemistry for the flowers (RDA, *P* = 0.034), but not for the leaves (RDA, *P* = 0.70). However, no consistency in chemical profiles explaining insect performance could be detected when comparing the two plant species or intra-specifically comparing leaves and flowers.

## Discussion

When female *P. brassicae* butterflies were presented with different developmental stages of *S. arvensis* or *B. nigra* plants, acceptance for oviposition decreased with plant age. Interestingly, selection of plants negatively correlated with plant size, i.e. the smallest plants were preferred for oviposition. On *S. arvensis* plants varying in age, oviposition preference of adult females correlated weakly with both larval development time and pupal mass. Caterpillars developed fastest and attained the largest biomass as pupae when they grew on the youngest plants, i.e. plants in the vegetative /early reproductive stage. No such correlation was found for *B. nigra*. Caterpillars developed into heavier pupae on *B. nigra* plants in the vegetative and mid-reproductive stage and were lighter on plants in the early reproductive stage, whereas there was no effect on development time. Chemical analyses of glucosinolates, amino acids and sugars in leaf and flower tissues collected from the two plant species in different developmental stages revealed that the differences in the analyzed plant metabolites were more evident with respect to tissue type than among different developmental stages or whether plants were damaged or not. Although for both plants species correlations were found between insect performance and plant chemistry variables, we did not detect clear consistencies in phytochemistry characteristics and insect performance.

Host plant selection for ovipostion sites by adult insect herbivores is a complex behavioral process that is guided by various sensory inputs (Carrasco et al. 2015; Finch and Collier 2000). Female butterflies select host plants for oviposition based on visual, olfactory and gustatory information (Bruce et al. 2005; Schoonhoven et al. 2005). In some species, concentrations or proportions of particular chemical compounds determine oviposition preference of female butterflies (Thompson and Pellmyr 1991). Caterpillars of *P. brassicae* are specialist feeders on plants containing glucosinolates, mainly species in the family Brassicaceae (Feltwell 1982). Studies have shown that glucosinolates stimulate oviposition by adult females and feeding by the larvae, respectively, although not all glucosinolate compounds do this to the same extent (Renwick et al. 1992; van Loon et al. 1992). Previous studies have shown that host plant recognition for oviposition by *P. brassicae* butterflies requires physical contact with the plant and is based on chemoreception of glucosinolates on the leaf surface (Renwick 2002; van Loon et al. 1992). On the leaves, where *P. brassicae* females habitually lay their eggs, concentrations of the dominant glucosinolate only decreased with plant age in *S. arvensis* and not in *B. nigra.* Van Loon et al. (1992) found that glucobrassicin is a much stronger oviposition stimulant than sinigrin, the dominant glucosinolate in *B. nigra*. The concentration of glucobrassicin was found to be very low in undamaged foliage of *B. nigra* and *S. arvensis* (< 0.8 and <0.06 μmol g^−1^ DW, respectively) and it did not change with plant development. Thus, sinigrin and glucobrassicin concentrations do not explain oviposition preference in relation to plant age in this study. Detection of glucosinolates by *P. brassicae* butterflies occurs most likely at the surface of the leaf. We do not know how whole-leaf glucosinolate content relates to that at the leaf surface.

Females of *P. brassicae* typically lay eggs in clutches varying between 10 and 50, although more than 100 eggs may be laid during one oviposition event. Depending on the number of caterpillars and the size of the food plants, several plants growing in close proximity are needed for the successful development of *P. brassicae* larvae from a single egg clutch. Thus both quantitative and qualitative characteristics of the food plant determine development and survival of *P. brassicae* caterpillars (Fei et al. 2016a). One area that is rarely considered in studies of oviposition behavior and plant preference in herbivores, however, is that the performance of offspring is not necessarily determined by current plant quality and quantity but by the ways in which these parameters are affected in the future. The plant is not a static resource during insect development, but is also growing and developing itself. Depending on local abiotic conditions like temperature, the entire egg-to-pupa development period may cover a month or more. Furthermore, as in most holometabolous insects, > 80% of insect feeding and growth in *P. brassicae* occurs during the final (= L5) instar. This stage can occur some 3–4 weeks after oviposition, meaning that the mother may have to be able to roughly predict plant quality (and quantity) several weeks in advance when selecting a plant on which to oviposit. Given that their short-lived food plants may be present for only 2–3 months in the field, this represents a significant fraction of the plant’s lifetime. *P. brassicae* may select plants not on the basis of present quality or quantity but more importantly on anticipating future quality and quantity coinciding with the final instar of their larvae. Laying eggs on plants in the mid to late reproductive phase may have serious consequences for offspring fitness and survival as plant tissues may be senescing by the time the larvae reach their final instar. Female butterflies may determine growth potential of fast growing annual plants based on chemical, visual and tactile sensory input or a combination of these.

Ontogenetic variation in primary and secondary metabolites may also influence the performance of insects feeding on plants differing in age (Quintero et al. 2014). In general, insects tend to prefer, have higher densities, and develop better on younger than on older plants (Boege and Marquis 2005). The developmental stage of the plants in this study had relatively small effects on *P. brassicae* larval performance variables such as development time and pupal mass and survival to pupation. Comparing chemical profiles of leaf and flower tissues collected from plants in different developmental stages revealed that differences in plant chemistry were more pronounced with respect to tissue type than to developmental stage. In addition to ontogenetic changes in chemistry, concentrations of plant phytochemicals also change in response to herbivory (Karban and Baldwin 1997) and herbivore-induced plant responses may also depend on plant ontogeny (Barton and Koricheva 2010; Quintero and Bowers 2011; van Dam et al. 2001). No consistencies were found for the two plant species in plant age-related and herbivore-induced changes in metabolite concentrations. Moreover, correlation analysis of insect performance and plant chemistry also did not reveal consistent patterns explaining insect performance in relation to plant chemistry. We only measured a selected number of chemical variables in the plant. Plant quality as food for caterpillars is not restricted to the compounds analyzed in this study. Phosphorous, lipids, ratios of certain amino acids / proteins, digestibility reducing enzymes, cellulose content, and the presence of other plant secondary metabolites may affect digestibility of the ingested food and contribute to the overall nutritional quality of the food plant (Speight et al. 2008). However, the results of this study suggest that if other plant nutritional traits varied in relation to plant age, their effects on larval performance were limited, especially in *B. nigra*.

Whereas young larvae of *P. brassicae* feed only on leaf tissues, older caterpillars move up the plant and prefer to feed on the flowers when present (Smallegange et al. 2007). Moreover, caterpillars that only had access to flowers developed faster and attained a greater final larval mass than conspecifics that only had access to *B. nigra* leaves (Smallegange et al. 2007). In our experiments, we allowed the caterpillars to move and feed freely on the leaves and the flowers. The mixed diet of the caterpillars may further explain the poor correlation between phytochemistry and insect performance. Nevertheless, it is notable that despite the large differences in chemistry between the leaves and the flowers, pupal mass and larval development time were relatively little affected. For example, the performance of caterpillars was similar on young *B. nigra* plants that had not yet produced flowers, and old *B. nigra* plants that had developed flowers. The discrepancy between the results reported in Smallegange et al. (2007) and this study can also be explained by the fact that larval mass (Smallegange et al. 2007) and pupal mass (this study) are not necessarily positively correlated (Ansari et al. 2012). Moreover, in the study by Smallegange et al. (2007) a single age class of *B. nigra* plant was used. *Pieris brassicae* has evolved to feed on a range of short lived annual species that all contain glucosinolates but also exhibit considerable variation in their chemical profiles, both quantitatively and qualitatively. The variation in plant quality among the plants used in this study may have been too small to strongly impact on larval performance (see also Gripenberg et al. 2010).

One critical point that is often ignored when studying behavior is that preference may be reduced in insect colonies that have been reared under standardized conditions in the laboratory, especially with respect to food quality. However, we found that female butterflies were still ‘choosy’ with respect to oviposition decisions. Cultures of our butterflies are partially replenished every summer, thus mitigating this effect. Moreover, in a recent study, we also found that oviposition preference of adult *P. brassicae* butterflies changed when they had developed as caterpillars on different host plant species (Fei et al. 2016b). Interestingly, these butterflies never preferred to oviposit on the plant species on which they had developed as caterpillars (Fei et al. 2016b). In combination, these results reveal that *P. brassicae* retains selectivity even when reared in the laboratory.

According to the ‘preference-performance’ hypothesis, insect herbivores should lay their eggs on host plants that are most suitable for development and survival of their offspring (Jaenike 1978). However, the results of studies on the relationship between adult oviposition preference and larval performance do not always corroborate this hypothesis (Gripenberg et al. 2010; Jaenike 1990; Mayhew 2001; Thompson and Pellmyr 1991;). Fitness benefits associated with host plants may differ for the adult mothers and her offspring (Scheirs et al. 2005). Oviposition preference and larval performance may be poorly correlated when the larval host plant is bad for the mother (e.g. when physical characteristics hamper egg laying, but not larval feeding), when there is variability in host-plant availability and quality in time and space, and when food plants are chosen by the offspring (Mayhew 2001; Thompson 1988; Wiklund and Friberg 2009). Other ecological factors, such as reduced risk of attack by the herbivore’s natural enemies, or enemy free space (Mira and Bernays 2002; Oppenheim and Gould 2002) and interactions with mutualists preventing predation (Atsatt 1981) may also play important roles when selecting plants for oviposition. Moreover, female physiology (age, egg loads) and optimization of survival of the eggs rather than the larvae (Tammaru et al. 1995) may further explain why adult preference and offspring performance are not necessarily positively correlated. Both *B. nigra* and *S. arvensis* have also been reported to respond to *P. brassicae* egg deposition by forming necrotic tissues, which negatively impacts survival of the eggs and growth and development of the caterpillars developing from these eggs (Hilker and Fatouros 2015; Pashalidou et al. 2015). Generally, inducibility of plant responses to herbivory tends to decrease with plant age (Barton and Koricheva 2010). In *B. nigra,* the response to egg deposition was also reduced in older plants (D. Lucas-Barbosa, unpublished data), but this did not appear to affect oviposition preference of *P. brassicae* in this study. As an alternative strategy, we propose that oviposition decisions may be based on future and not present plant quality. The biology of the plant, in terms of the rate of growth and dynamic changes in primary and secondary chemistry may also depend on life-history traits of the plant, e.g., whether the plant is a long-lived perennial or a short-lived annual. Thus preference-performance studies need to account for differences in the biology of the food plants and the biology of the herbivore.

In summary, our study reveals that adult *P. brassicae* female butterflies distinguish between different developmental stages of their food plants and prefer younger stages of both *S. arvensis* and *B. nigra* for oviposition, whereas the consequences of these preferences for larval growth and development were relatively small. In the Netherlands *S. arvensis* and *B. nigra,* which often grow in large stands, are important food plant species for *P. brassicae.* For a multivoltine insect herbivore like *P. brassicae* that primarily feeds on short-lived annuals, it is a challenge for different generations to locate suitable host plants. Preference for younger host plants may allow the plant to grow additional resources to support growth of the larvae that feed gregariously and are particularly voracious during the final instar. For a multivoltine insect that feeds on short-lived, fast growing plants it is also adaptive to be able to exploit a broad range of food plants and to be little affected by ontogenetic changes in plant quality.

## Electronic supplementary material

ESM 1 (PDF 433 kb)
