# Supplementary material for: Oviposition Preference for Young Plants by the Large Cabbage Butterfly (Pieris brassicae) Does not Strongly Correlate with Caterpillar Performance
Source: J Chem Ecol. 2017 Jun 15;43(6):617–29. doi: 10.1007/s10886-017-0853-9 (PMC5501907; doi:10.1007/s10886-017-0853-9)
Supplement: Supplementary file 1 — (PDF 433 kb) [file 10886_2017_853_MOESM1_ESM.pdf]

PREFERENCE FOR YOUNG PLANTS BY CABBAGE BUTTERFLY (*Pieris brassicae*) MOTHERS DOES NOT STRONGLY CORRELATE WITH OFFSPRING PERFORMANCE

MINGHUI FEI[1], JEFFREY A. HARVEY[1,2], YI YIN[1], AND RIETA GOLS[3*]

[1]*Netherlands Institute of Ecology, Department of Terrestrial Ecology, Wageningen, the Netherlands*

[2]*VU University Amsterdam, Department of Ecological Sciences, Section Animal Ecology, Amsterdam, the Netherlands*

[3]*Laboratory of Entomology, Wageningen University, Wageningen, the Netherlands*

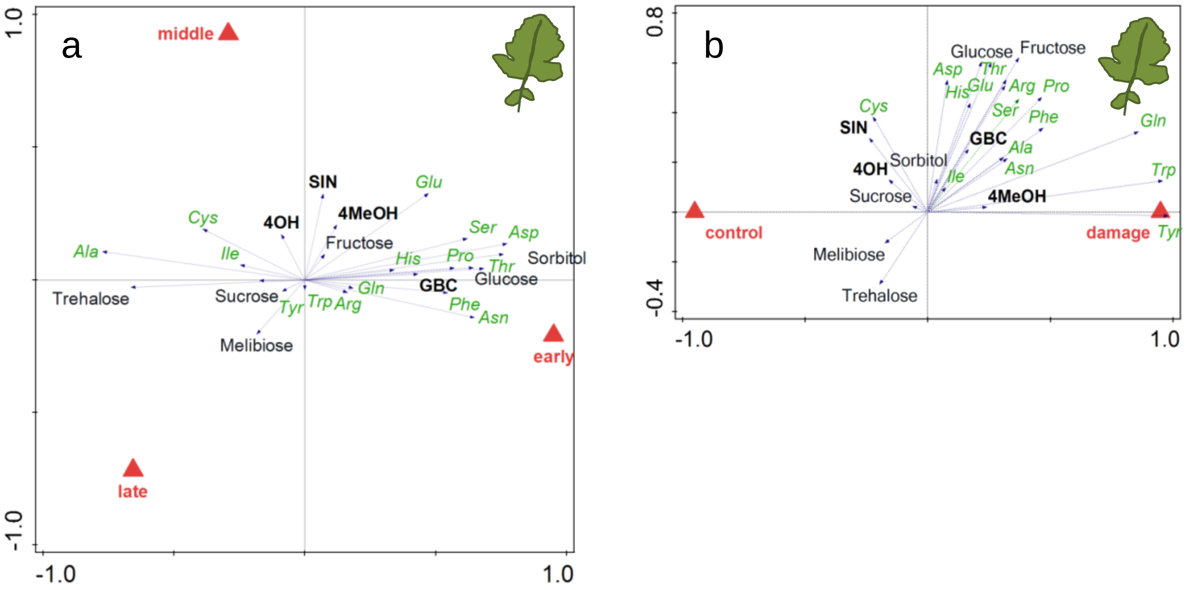

Figure S1 RDA analyses showing phytpchemical separation of plant developmental stages (a) and damage status (b,) of *Brassica nigra* leaves. The centroids (red triangles) depict mean scores for the various classes. The loadings (contribution of the chemical variables) are given in dotted lines and arrows with the names of the glucosinolate in bold black font, amino acids in italicized green font and the sugars in black normal font. The distance from the axes origin to each of the arrows indicates the magnitude of the contribution of the variable to the principal components. For the amino acids,the international IUPAC three letter codes were used. Glucosinolate abbreviations: SIN = sinigrin, 4OH = 4-hydroxyglucobrassicin, GBC = glucobrassicin, 4MeOH = 4-methoxyglucobrassicin. Early represents early-vegetative phase, middle represents late-vegetative/early reproductive phase, and late represents middle-reproductive phase.

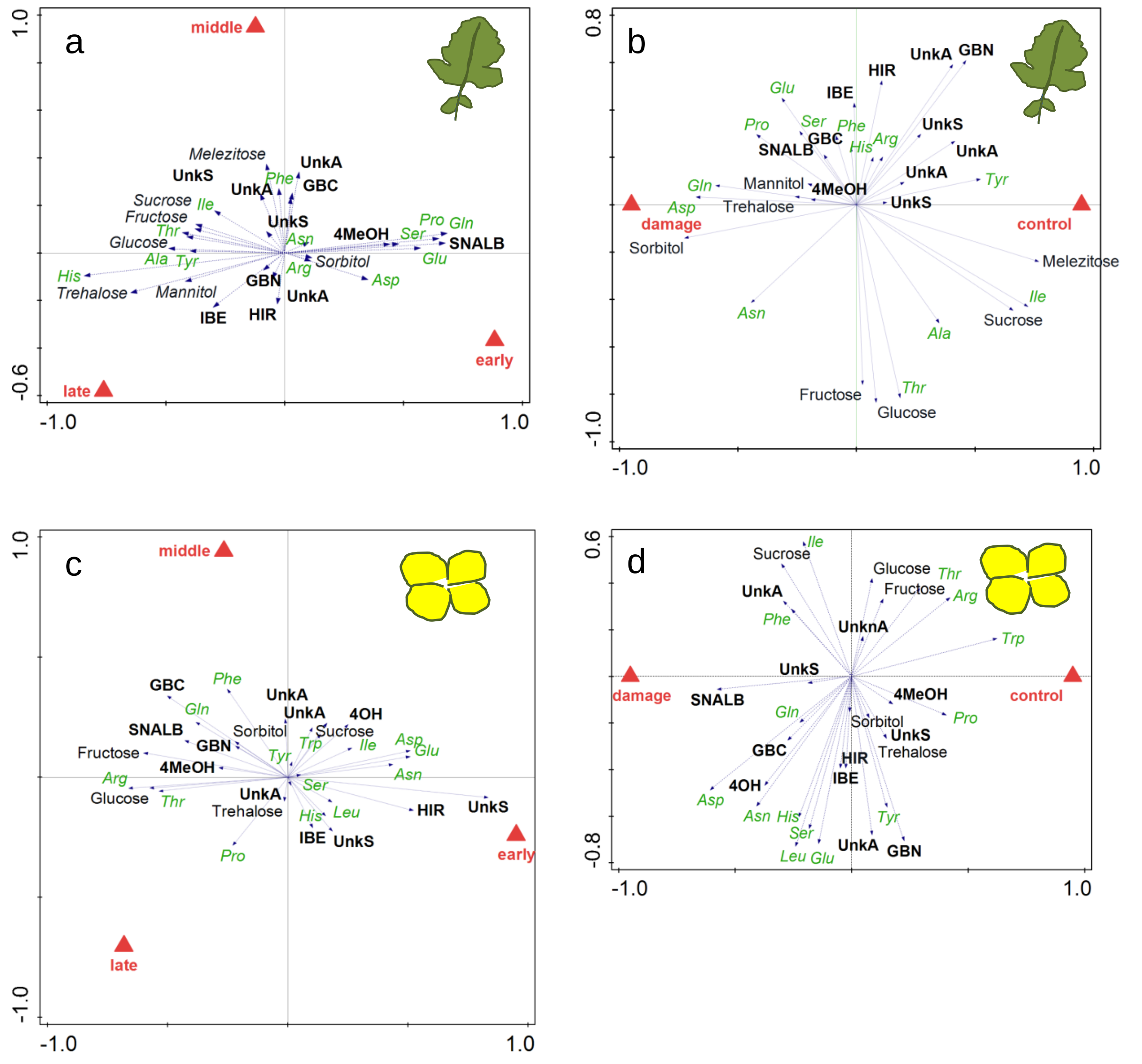

Figure S2 RDA analyses showing phytpchemical separation of plant developmental stages (a, c) and damage status (b, d) of *Sinapis arvensis* leaves (a, b) and flowers (c, d). The centroids (red triangles) depict mean scores for the various classes. The loadings (contribution of the chemical variables) are given in dotted lines and arrows with the names of the glucosinolate in bold black font, amino acids in italicized green font and the sugars in black normal font. The distance from the axes origin to each of the arrows indicates the magnitude of the contribution of the variable to the principal components. For the amino acids,the international IUPAC three letter codes were used. Glucosinolate abbreviations: GBN = glucobrassicanapin, IBE = glucosiberin, HIR = glucohirsutin, UnkS = unkown sulfur containing glucosinolate, UnkA = unkown aliphatic GS (not further specified). 4OH = 4-hydroxyglucobrassicin, GBC=glucobrassicin, 4MeOH = 4-methoxyglucobrassicin SNALB = sinalbin. Early represents late-vegetative/early reproductive phase, middle represents mid-reproductive phase, and late represents late-reproductive phase.
